# A Pilot Survey on Hygienic–Sanitary Characteristics of Ready-To-Eat Sauces and Pesto

**DOI:** 10.3390/ijerph17145005

**Published:** 2020-07-12

**Authors:** Giuseppina Caggiano, Giusy Diella, Paolo Trerotoli, Marco Lopuzzo, Francesco Triggiano, Massimo Ricci, Vincenzo Marcotrigiano, Maria Teresa Montagna, Osvalda De Giglio

**Affiliations:** 1 Department of Biomedical Science and Human Oncology Hygiene Section–University of Bari Aldo Moro, Medical School, Piazza G. Cesare 11, 70124 Bari, Italy; giusy.diella@uniba.it (G.D.); paolo.trerotoli@uniba.it (P.T.); marcolopuzzo@gmail.com (M.L.); francesco.triggiano@uniba.it (F.T.); osvalda.degiglio@uniba.it (O.D.G.); 2 ARPA Puglia, Regional Agency of the Environmental Prevention and Protection, Department of Brindisi Operative Unit of Food and Drink, via Galanti, 16, 72100 Brindisi, Italy; m.ricci@arpa.puglia.it; 3 Department of Prevention, Food Hygiene and Nutrition Service, Local Health Unit BT, Barletta-Andria-Trani, 76125 Trani, Italy; vincenzo.marcotrigiano@aslbat.it

**Keywords:** ready-to-eat food, sauces and pesto, foodborne diseases, public health, microbiological analysis, fungi

## Abstract

In recent years, the chaotic habits of modern life have favored the consumption of quickly prepared meals, using ready-to-eat (RTE) foods and condiments. The aim of this study was to establish the microbiological safety of RTE sauces and pesto from markets analyzed at different stages of shelf life. In the bacterial investigation, all samples were shown to be acceptable, although differences were observed concerning shelf life times. On the other hand, the fungal investigation showed frequent positive results, with concentrations higher than threshold values. Detected microbial diffusion was the lowest when products were far from the expiry date and had just been opened, while high microbial proliferation was observed when analyzing the same package after 48 h, higher than for a product close to the end of its shelf life. This study highlights the discreet microbiological quality of processed and RTE foods, underlining the importance of hygienic–sanitary surveillance of these foods to their shelf life. Consequently, it is necessary to: (1) implement a food control plan for all food categories to carry out risk analysis associated with their consumption; and (2) better adapt the regulations relating to microbiological analysis, and understand the biological significance of each microbial parameter throughout the shelf life of foods.

## 1. Introduction

Foodborne diseases (FBDs) are a crucial and growing public health problem worldwide [1], causing significant economic losses and medical costs. Also known as food poisoning, these diseases encompass a wide spectrum of illnesses caused by the ingestion of contaminated foods. The contamination can be caused by different germs (bacteria, viruses and parasites) or chemical agents, and can be linked to environmental contamination, including pollution of water, soil and air. To date, researchers have identified more than 250 FBDs. The Center for Disease Control and Prevention (CDC) estimates that 48 million people get sick from FBDs every year, while 128,000 are hospitalized and 3000 die [2]. According to the latest published data reported by European Union (EU) Member States, in 2018, 5146 foodborne outbreaks affected 48,365 people. Salmonellosis was the second-most commonly reported gastrointestinal infection in humans in the EU (91,857 cases reported) after campylobacteriosis (246,571 cases); Slovakia, Spain and Poland accounted for 67% of the 1581 salmonellosis cases, mainly linked to egg consumption [3].

Although the food of animal origin is the main source of documented FBD outbreaks, the number of human cases associated with food of non-animal origin appears to be increasing [4,5].

In recent years, the chaotic habits of modern life have favored the consumption of quickly prepared meals, using ready-to-eat (RTE) foods and condiments, that are capable of satisfying any gastronomic needs. The consumption of mixed salads with vegetables and different ingredients has also increased [6].

As is the case with other RTE foods, salads—often intended as whole meals—are readily available in the refrigerated sections of supermarkets, packaged in plastic bowls [4]. Although current food technologies, innovative packaging systems and the extension of product shelf life contribute to guaranteeing the healthfulness of products intended for increasingly attentive and demanding consumers, it is still difficult to achieve absolute microbiological safety in the food sector. Consequently, the hygienic–sanitary surveillance of RTE foods, especially those with multiple ingredients, is undoubtedly useful for the prevention of infections.

European Commission Regulation No. 852/2004 on the hygiene of foodstuffs provides a risk-based approach to controlling food hygiene [7,8]. The regulation requires food business operators to implement a food safety management system, based on hazard analysis and critical control point (HACCP) principles, to maintain good hygienic conditions in the premises and ensure that food staff are trained in good hygiene practices [8]. Concerning microbiological aspects, perishable and RTE food matrices are considered worthy of attention, given their intrinsic conditions favoring microbial proliferation compared to the nutrient capacity of the substrate.

The responsible microorganisms more often found in RTE foods with vegetables are viruses (norovirus and hepatitis A), parasites (*Cryptosporidium* spp., *Giardia* spp. and *Toxoplasma gondii*) and bacteria (*Salmonella* spp., enterotoxigenic *Escherichia coli* (ETEC), *Campylobacter* spp., pathogenic *Yersinia enterocolitica* and *Listeria monocytogenes*) [5,9].

There are reports in the literature on FBD outbreaks resulting from the consumption of pesto—a condiment made with basil, oil and sometimes garlic, typical of Liguria (North Italy)—such as an ETEC outbreak in Denmark [10] and outbreaks of cyclosporiasis [11]. The research, and therefore understanding regarding the underlying causes of these outbreaks is still poor, and represents a barrier to effectively addressing this issue. Furthermore, the use of rapid and accurate detection of foodborne pathogens is an international priority to control and prevent foodborne epidemics in humans, and to reduce mortality rates drastically [12].

To date, there are no Italian regulations regarding the microbiological contamination of RTE foods. The aim of this pilot study was to investigate the microbiological safety of RTE sauces and pesto collected from markets and analyzed at different stages of shelf life, evaluating various microbial parameters that are relevant to consumer health.

## 2. Materials and Methods

### 2.1. Study Design

Forty-five samples (14 cheese sauce, 13 basil pesto, 13 pore mushroom sauce and five walnut sauce) were randomly collected from numerous retail stores located in southern Italy. These markets were registered in accordance with EC Reg. 852/2004 [7], and selected their suppliers in order to guarantee safe foodstuffs to consumers. Taking into account the steps of the whole food chain, producers and retailers, each for their own competence, must comply with the sector hygiene prerequisites, guarantee good hygiene and manufacturing practices (GHP, GMP) and apply the provisions of own-check control plans drawn up according to the principles of the HACCP system. Two packages corresponding to each production batch were collected for the same brand, for a total of 90 packs, with weight ranging from 140 to 200 g.

Fresh sauces with a shelf life (the date until which the foodstuff retains its specific properties when properly stored) of three months, to be consumed within four days of opening the package, keeping it at 0–4 °C, as reported on the label of three months, which had not undergone sterilization heat treatment, were also analyzed. Samples were packaged in a protective atmosphere and stored in the dark at low relative humidity and under refrigeration (+4 °C) until before analysis.

Only samples without garlic and chili were collected, in order to avoid its documented potential antibacterial effect influencing the results [13,14,15].

The analysis was carried out in three stages of each sample’s shelf life. The first package was subjected to analysis at the time of opening (T0) and then two days after opening (T1), keeping the sample at +4 °C. The second package of the same lot (remained sealed) was analyzed two days before the manufacturer’s expiry date (T2).

### 2.2. Bacteria Investigation

Sauce samples were examined for mesophilic microorganisms (Enterobacteriaceae, *Escherichia coli*, *Bacillus cereus*, positive-coagulase *Staphylococcus*, *Clostridium perfringens*, *Listeria monocytogenes*, *Salmonella* and *Listeria monocytogenes*) according to the standardized methods detailed below. The microbiology results were interpreted with the microbiological criteria of some RTE foods found in the literature [8,16]. Samples were considered non-compliant if they exceeded the acceptable value.

For the quantitative analysis, 10 g of each sample was added to 90 mL of Buffered Peptone Water (Biolife Italiana srl, Milano, Italy), homogenized in a stomacher (Biosigma, Cona, Verona, Italy) for 1 min and subject to three rounds of 10-fold serial dilution.

To count mesophilic microorganisms, 1 mL of each dilution was inoculated by the inclusion technique on Plate Count Agar (PCA; Microbiol S.n.c., Cagliari, Italy) and incubated at 30 °C for 72 h [17]. Samples with microbial counts of ≤10^6^ colony forming units/gram (cfu/g) were considered satisfactory, while samples with counts between 10^6^ and <10^7^ cfu/g were considered acceptable [8,16].

The enumeration of Enterobacteriaceae was performed using the inclusion technique with Violet Red Bile Glucose Agar (VRBG; Microbiol S.n.c., Cagliari, Italy) and incubation at 37 °C for 24 h [18]. Typical pink or red-violet colonies with or without halos were inoculated on a nutrient agar (Biolife Italiana srl, Milano, Italy) for 24 h at 37 °C, and underwent a confirmatory biochemical test with an API (Analytical Profile Index) of 20E (Biomèrieux, Marcy l’Etoile, France) [18]. Enterobacteriaceae counts of <10^3^ cfu/g and between 10^3^ and <10^4^ cfu/g were considered satisfactory and acceptable, respectively [8,16].

The enumeration of *E. coli* was performed using the inclusion technique with a Tryptone Bile X-glucose (TBX) agar (Microbiol S.n.c., Cagliari, Italy) and incubation at 44 °C for 24 h [19]. Blue colonies were identified as *E. coli* [16]. *E. coli* counts of <10^4^ cfu/g and between 10^4^ and <10^5^ cfu/g were considered satisfactory and acceptable, respectively [8,16].

For evaluation of *Bacillus cereus*, samples were inoculated on a Mannitol Egg Yolk Polymyxin (MYP) agar (Liofilchem S.r.l, Roseto degli Abruzzi, Italy) and incubated at 30 °C for 24–48 h [20]. Suspected blue colonies were transferred to Columbia Agar (Biomèrieux, Marcy l’Etoile, France) and incubated at 37 °C for 24 h. *B. cereus* was identified by the presence of typical haemolysis halos [20]. *B. cereus* counts of <10^2^ cfu/g and between 10^2^ and <10^3^ cfu/g were considered satisfactory and acceptable, respectively [8,16].

The number of coagulase-positive *Staphylococcus* (later referred to as *Staphylococcus aureus*) was evaluated by inoculating the samples on a Baird-Parker agar (Biolife Italiana srl, Milano, Italy) [21]. After 48 h at 37 °C, typical colonies (black and grey, shiny and convex, surrounded by a zone of clearing in the medium) were transferred in a Brain Heart Infusion Broth (Biolife Italiana srl, Milano, Italy) and incubated at 37 °C for 24 h for subsequent coagulase testing with rabbit plasma (Liofilchem S.r.l, Roseto degli Abruzzi, Italy) [21]. Strains were identified at the species level by biochemical tests using an API Staph (Biomèrieux, Marcy l’Etoile, France). *S. aureus* counts of cfu/g <10^2^ and between 10^2^ and ≤10^3^ cfu/g were considered satisfactory and acceptable, respectively [8,16].

For enumeration of *Clostridium perfringens*, the samples were inoculated on a Tryptose Sulphite Cycloserine (TSC) agar (Biolife Italiana srl, Milano, Italy) and incubated at 37 °C for 24 h under anaerobic conditions [22]. Black colonies were subject to further biochemical testing for identification (acid phosphatase; Biolife Italiana srl, Milano, Italy) [22]. *C. perfringens* counts of <10 cfu/g and between 10 and ≤10^2^ cfu/g were considered satisfactory and acceptable, respectively [8,16].

For evaluation of *L. monocytogenes,* 25 g of each sample was inoculated in 225 mL of pre-enrichment Half-Fraser Broth (Biolife Italiana srl, Milano, Italy). After incubation at 30 °C for 24 h, 100 µL was transferred to Fraser Broth (Biolife Italiana srl) and incubated at 37 °C for 24 h [23]. Each enrichment medium was plated on an Ottaviani and Agosti listeria agar (ALOA; Microbiol S.n.c., Cagliari, Italy) and Palcam agar (Biolife Italiana srl, Milano, Italy) [23]. After incubation at 37 °C for 24–48 h, the colonies were inoculated into a Columbia agar before proceeding to the confirmation test [23]. No detection in 25 g was considered satisfactory. Biochemical tests to identify *Listeria* species were conducted using API Listeria (Biomèrieux, Marcy l’Etoile, France) [8,16].

For enumeration of *L. monocytogenes*, samples were inoculated in plates with ALOA and incubated at 37 °C for 24–48 h. Suspected blue colonies were transferred to a Columbia agar (Biomèrieux, Marcy l’Etoile, France) at 37 °C for 24 h to observe the presence of haemolysis, and at the same time, xylose and rhamnose tests (Liofilchem S.r.l, Roseto degli Abruzzi, Italy) were performed [24].

For the investigation of possible *Salmonella*, 25 g of each sample was added to 225 mL of Buffered Peptone Water pre-enrichment medium (Biolife Italiana srl, Milano, Italy) [25]. After incubation at 37 °C for 24 h, 100 µL of the sample was transferred to a Modified Semisolid Rappaport Vassiliadis (MSRV) agar selective enrichment medium (Microbiol S.n.c., Cagliari, Italy) and incubated at 41.5 °C for 24 h, and 1 mL was transferred to the Muller–Kauffmann Tetrathionate–Novobiocin Broth (MKTTn, Biolife Italiana srl, Milano, Italy) at 37 °C for 24 h [25]. Then, the enrichment was inoculated into a Xylose Lysine Desoxycholate (XLD) agar specific selective culture medium (Biolife Italiana srl, Milano, Italy) and Salmonella–Shigella (SS) agar (Biomèrieux, Marcy l’Etoile, France) and incubated at 37 °C for 24 h and 24–48 h, respectively. Typical black-centered colonies underwent further biochemical testing for identification (API 20E, Biomèrieux, Marcy l’Etoile, France) [25]. No detection in 25 g was considered satisfactory [8,16].

### 2.3. Fungi Investigation

An aliquot of 10 g of sample was inoculated in 90 mL of Buffered Peptone Water (Biolife Italiana srl, Milano, Italy) and homogenized in a stomacher (Biosigma, Cona, Verona, Italy) for 1 min. Three serial decimal dilutions were equipped, and 100 mcl was inoculated on a Dichloran Rose Bengal Chloramphenicol (DRBC) agar (Liofilchem S.r.l, Roseto degli Abruzzi, Italy) and incubated at 25 °C for five days. Yeast colonies were identified using a semi-automated sugar assimilation system (API ID 32C, Biomèrieux, Marcy l’Etoile, France), and filamentous fungi were identified, evaluating their standard macroscopic and microscopic characteristics [26]. As a reference value for fungi, a concentration of 10^3^ cfu/g was defined as the threshold value [27]. Samples exceeding the acceptability value were considered non-compliant.

### 2.4. Statistical Analysis

Bacteria, molds and yeasts were counted, and the mean values were used to evaluate differences according to substrata, applying a general mixed model in which data were assumed to have a Poisson distribution. Results are presented as means and 95% confidence intervals as estimated by the model, after back transformation from the link function (the natural logarithm of the counts). The model had time and package as fixed effects, and the type of sauce was a random effect. The post hoc comparison was adjusted according to Tukey, and the differences in results are shown as an estimated difference on a log scale and a 95% confidence interval (CI). To evaluate the type of sauce, results are shown as estimated covariance parameters of random effects, standard error (SE) and estimated intercepts with SE.

A value of *p* < 0.05 was considered to indicate statistical significance. The analysis was conducted using SAS 9.4 (Analytics Software and Solutions; Cary, NC, USA; version for personal use).

## 3. Results

### 3.1. Bacterial Investigation

The results for all 90 samples indicated they were either satisfactory or acceptable, with differences in the count observed for different shelf life times.

The mean bacterial counts for the first package were 61,609 cfu/g (95% CI: 22,418–169,311) at T0 and 83,617 cfu/g (95% CI: 30,426–229,793) at T1 (Table 1), corresponding to a difference of −0.305 (95% CI: −0.31 to −0.30, *p* < 0.0001) that was statistically significantly different. The bacterial count of the second package at T2 was 83,617 cfu/g (95% CI: 30,426–229,793). All differences with respect to the first package were statistically significant: for T2 vs. T0 the difference was −0.67 (95% CI: −0.68 to −0.68, *p* < 0.0001); and for T2 vs. T1 the difference was 0.367 (95% CI: 0.362–0.372, *p* < 0.0001). The covariance parameter for the bacterial count was 0.68 (SE = 0.48), so the variance among sauces was low, but estimates revealed higher counts for packages of mushroom sauce (intercept 1.2, SE = 0.41, *p* = 0.0269) and lower counts for walnut sauce (intercept −1.02, SE = 0.41, *p* = 0.048).

The *S. aureus* count was 132 cfu/g (95% CI: 40.72–433.35) for the first package at T0 and 25.3 cfu/g (95% CI: 7.75–82.56) at T1; the difference between these two times is 1.6 (95% CI: 1.6 to 1.7, *p* < 0.0001). The count for the second package at T2 was 7.5 cfu/g (95% CI: 2.3–24.6). This is significantly different from the first package: for T0 vs. T2 it was 2.87 (95% CI: 2.78–2.96, *p* < 0.0001) with a difference of −1.21 (95% CI: −1.31 to −1.12, *p* < 0.0001) comparing T1 vs. T2. The covariance parameter for the type of sauce (the random effect) was 0.93 (SE = 0.66). Only the intercept for mushroom was statistically significant (effect 1.6, SE = 0.48, *p* = 0.141). Biochemical tests confirmed the identity of the isolated strains as *S. aureus*.

Regarding *B. cereus*, the counts showed no differences between packages or according to time: the first package at T0 had a count of 3.76 cfu/g (95% CI: 2.1–6.8), and at T1 it was 4.32 cfu/g (95% CI: 2.39–7.82). The difference was not statistically significant (*p* = 0.1815). The count for the second package at T2 was 4.32 cfu/g (95% CI: 2.38–7.81), and did not significantly differ from the first package at T0 (*p* = 0.7711) or at T1 (*p* = 0.4224). The covariance parameter was 0.22 cfu/g (SE = 0.16), and the intercept for pesto sauce was statistically significant (intercept = 0.61, SE = 0.24, *p* = 0.44).

No Enterobacteriaceae, *E. coli*, *C. perfringens* or *Salmonella* spp. were found in any of the tested samples.

*L. monocytogenes* was also not isolated, but two pesto samples and one cheese sauce were contaminated with other *Listeria* species, such *L. welshimeri* in one sample of pesto, *L. innocua* in one mushroom sauce sample and *L. seeligeri* in one sample of cheese sauce, as found in all phases of analysis.

### 3.2. Fungi Investigation

Regarding yeasts, 22.2% of samples were non-compliant at T0 (eight basil pesto, one mushroom sauce and one walnut sauce), 28.9% at T1 (10 pesto, two mushroom sauce and one walnut sauce) and 35.6% at T2 (10 pesto, two mushroom sauce, two cheese sauce and two walnut sauce).

The mean for yeasts based on the first package was 2544.18 cfu/g (95% CI: 1063.3–6087.5) at T0 and 8326.45 cfu/g (95% CI 3479.9–19,923) at T1. The difference in the model estimate was −0.438 and was statistically significant (95% CI: −0.44 to −0.425, *p* < 0.0001). The estimated count for the second package at T2 was 3919.9 cfu/g (95% CI: 1638.3–9379.3), and was significantly different from the first package: for T2 vs. T0 it was −1.186 (95% CI: −1.192 to −1.179, *p* < 0.0001), while for T2 vs. T1 it was 0.75 (95% CI: 0.74–0.76, *p* < 0.0001).

The covariance parameter of the type of sauce for the model applied to yeasts was 0.51 (SE = 0.36), so there was not wide variance among types of sauce. The intercept for pesto was 0.92 (SE = 0.36), revealing higher counts in pesto, and was the only one that was slightly statistically significant (*p* = 0.0416). Table 2 shows the isolated yeast species.

Regarding molds, 2.2% of samples were non-compliant at T0 and T1 (one cheese sauce), and 8.9% at T2 (two mushroom sauce, one cheese sauce and one walnut sauce).

The mean for molds estimated by the model was 56.6 cfu/g (95% CI: 21.41–165.9) for the first package at T0 and 183.4 cfu/g (95% CI: 65.89–510.4) at T1. The difference of model estimate was −0.33 (95% CI: −0.37 to −0.28, *p* < 0.0001). For the second package at T2, the mold count was 82.6 cfu/g (95% CI: 29.68–229.9). The difference between T2 and T0 was −1.12 (95% CI: −1.116 to −1.08, *p* < 0.0001), while between T2 and T1 it was 0.79 (95% CI: 0.76–0.3, *p* < 0.0001).

No large variances were observed among the types of sauce, given the value of the random effect of 0.69 (SE = 0.49), and only the intercept of pesto was statistically significant (estimate effect: −1.36, SE = 0.42, *p* = 0.017). *Aspergillus* spp. and *Penicillium* spp. were the most frequently isolated molds (Table 3).

## 4. Discussion

Food safety and consumer protection are a priority for the European Parliament; the publication of the White Paper on Food Safety and the regulations that have evolved over time have allowed the implementation and perfection of a control system, ranging from food production to food contact materials, from obligations regarding traceability to the Rapid Alert System for Food and Feed (RASFF) [7,28,29,30]. RASFF is the instrument that ensures the rapid transmission of information among the member states, and the recall and withdrawal of foods when food safety risks are detected. Despite advances in legislation, regulations relating to RTE foods are still lacking, especially for those with multiple ingredients. European Community legislation provides for the monitoring of the presence of *L. monocytogenes* and *Salmonella* among the food safety criteria for RTE foods [31], leaving out other microbiological parameters.

The study results here highlight a discreet microbiological quality of processed and RTE foods, with an increase of non-compliant products close to the expiry date. This underlines the importance of hygienic–sanitary surveillance of this type of food in relation to its shelf life. In particular, microbial contamination was shown to be low when far from the expiry date and upon opening of the package, while high microbial proliferation was observed when analyzing the same opened package after 48 h. In addition, the product had higher microbial proliferation when at the end of its shelf life, but this was lower than when the product had been opened for 48 h, even if properly stored. Relative to the types of microbial contaminants and the non-compliance of foods, our results show a prevalence of fungi compared to the other investigated microorganisms.

This finding is consistent with the totality of official surveillance data registered at RASFF over the past 10 years [32]. RASFF reported that out of 239 notifications on sauces, the majority (85.7%) referred to the presence of fragments, additives, chemicals, undeclared ingredients and others. Those relating to microbiological contamination include the presence of bacterial toxins (e.g., botulinum toxin), in which countries reported only 34 notifications. It is very interesting to note that among the notifications for microbiological contamination, 11/34 sauces (32.34%) were contaminated by fungi (yeasts or molds) [32].

In our study, the isolation of *Aspergillus flavus* is important to underline, as it represents one of the main species responsible for food contamination. It can produce aflatoxin B1 (AFB1) and B2 under favorable conditions (e.g., of temperature and relative humidity) and in improper storage conditions during transport and at stores [33]. Aflatoxins represent one of the main groups of mycotoxins produced in nature. It would be interesting to investigate their presence in these foods to protect the health of consumers from AFB1, a highly dangerous contaminant classified as a human carcinogen (group 1) by the International Agency for Research on Cancer [34].

The detected yeast species were environment-related, and their presence in food is not atypical. Foodborne yeasts, excluding yeasts used as starter cultures, are commonly considered as food spoilage microorganisms [35]. However, some strains, such as *C. lusitaniae*, *C. famata*, *C. krusei*, *C. colliculosa*, *C. parapsilosis* and *C. tropicalis*, which show a relevant similarity to clinical *C. albicans*, are allocated to a group of potentially risky pathogens [35].

*B. cereus* and *S. aureus* are considered the most common pathogens of FBDs in developed and developing countries. In the period 2009–2015, the United States reported 5760 outbreaks, including 100,939 illnesses, 5699 hospitalizations and 145 deaths [36,37]. *B. cereus* is ubiquitous in the environment, and can be found in food and food-contact materials [38]. Spores can survive the mild heat treatments given to foods; moreover, *B. cereus* can develop at 8 °C and below, posing a danger to human health by the consumption of minimally or inappropriately processed chilled foods [38]. *B. cereus* toxins also pose a hazard to consumers because they can lead to serious forms of foodborne illnesses [38]. Foods associated with diarrheal food poisoning include soups, sauces and stews, meat products, fish, poultry, milk and dairy products and vegetables [38,39]. In our pilot study, according to the fixed threshold value, we did not detect a high bacterial concentration of *B. cereus*, and therefore the tested foods did not show a considerable risk of exposure to this microorganism.

*S. aureus* is a commensal and opportunistic pathogen, and its infection and colonization of livestock and farm workers have been described as possible sources of food contamination [40]. Considering that foodborne *S. aureus* illness is one of the most frequently reported diseases worldwide, its presence among food workers represents a public health risk, and workers have to put in place proper hygiene and control measures [40].

Considering *L. monocytogenes*, even if none of our samples were considered non-compliant, different species were isolated. *L. innocua* was initially considered non-pathogenic (hence its name) [41]. It also was considered non-haemolytic, but recent work has identified a number of haemolytic *L. innocua* isolates, which suggests that some isolates had presumably been (mis-)classified as *L. monocytogenes* [41]. Interestingly, some haemolytic *L. innocua* have been shown to invade (human) Caco-2 cells at the same levels as *L. monocytogenes* [41,42], while another study characterized the haemolytic *L. innocua* strain PRL/NW 15B95 as avirulent in a mouse model [41,43]. *L. innocua* has also been isolated at least once from a fatal human case, further supporting that at least some *L. innocua* may be able to cause human disease [41,44].

Reports in the literature show that the presence of *L. innocua* could mask *L. monocytogenes* and lead to false negative results in research on *L. monocytogenes* [45]. When *L. innocua* is present in the test sample, recovery of *L. monocytogenes* becomes problematic, especially since the two species have similar colony morphologies on isolation media. The use of species differentiating chromogenic media during streak plate isolation can help in the selection, but only if the population differential between *L. innocua* and *L. monocytogenes* is relatively small; if the population differential is large, then the resulting isolated colonies will all be the predominant species, which is typically *L. innocua* [46,47]. According to Dailey et al. [47], other non-pathogenic species of *Listeria* such as *L. welshimeri* can complicate the recovery of *L. monocytogenes*, suggesting that competition during selective enrichment is not limited to the presence of just *L. innocua*.

Although the latest RASFF annual report, published in 2019, documented a foodborne outbreak of *Salmonella agona*, possibly linked to RTE food in the UK in 2018 [32], from our analysis, *Salmonella* was not found as a contaminant of sauces and pesto.

Besides *Salmonella*, neither Enterobacteriaceae, *E. coli* nor *C. perfringens* were found, but this must not lead to underestimating the antibacterial power of some ingredients of the pesto and sauces examined. Although a parameter for the selection of the examined foods was the absence of garlic, a food with demonstrated antibacterial activity [12,13], numerous scientific studies also demonstrated the antibacterial power of basil essential oil against these microorganisms [48,49] and of pine nuts [50], which are included in pesto; walnuts [51] and pore mushroom [52] also showed antibacterial properties.

According to the latest Eurobarometer, less than one-third of European citizens rank food poisoning from bacteria among their top five concerns regarding food safety. The number of reported outbreaks suggests that there is an opportunity to raise awareness among consumers, as many foodborne illnesses can be prevented by improving hygiene measures when handling and preparing food [3].

## 5. Conclusions

Our study highlights the discreet microbiological quality of processed and RTE foods. The existence of RASFF notifications for sauces over the years demonstrates that these foods are not free from contamination, and are even sometimes contaminated by potentially lethal organisms such as botulinum toxin.

Considering that molds and yeasts in sauces are the most frequent contaminants, it would be advisable to assess regulations for fungi in this regard. It would be appropriate to place a greater emphasis on trying to better understand the role of fungi in food deterioration and their importance in defining product quality, identifying them at the genus and species level, and setting compliance threshold values for the whole life of commercial products.

It is necessary to implement a national food monitoring control plan for all food categories, and search for more parameters in order to conduct a risk analysis associated with their consumption. This would allow for better adaptation to the regulations related to microbiological analysis, and a better understanding of the biological significance of each microorganism throughout the shelf life of food.

## Figures and Tables

**Table 1:** Microorganism counts in analyzed samples, reported as mean and 95% confidence interval and relative differences.

	Microorganism Count (cfu/g (95% CI))		
Microbiologic parameter	T0	T1	T2
Mesophilic microorganisms	61,609 (22,418–169,311) *	83,617 (30,426–229,793)	83,617 (30,426–229,793) ’
*B. cereus*	3.76 (2.1–6.8)	4.32 (2.39–7.82)	4.32 (2.38–7.81)
Positive-coagulase *Staphylococcus*	132 (40.72–433.35) *	25.29 (7.75–82.56)	7.52 (2.3–24.6) ’
Molds	56.61 (21.41–165.9) *	183.39 (65.89–510.4)	82.63 (29.68–229.9) ’
Yeasts	2544.18 (1063.3–6087.5) *	8326.45 (3479.9–19,923)	3919.9 (1638.3–9379.3) ‘

cfu/g, colony forming units/gram; CI, confidence interval. * *p* < 0.05 T0 vs. T1; ’ *p* < 0.05 T2 vs. T0 and T2 vs. T1. Enterobacteriaceae, *E. coli*, *C. perfringens*, *L. monocytogenes* and *Salmonella* are not reported because they were absent in all analyzed samples.

**Table 2:** Yeast species isolated in cheese sauce, basil pesto, mushroom sauce and walnut sauce samples.

Isolated Yeasts	Samples (No.)			
	Cheese Sauce (14)	Basil Pesto (13)	Mushroom Sauce (13)	Walnut Sauce (5)
	% Contaminated Samples (No.)			
*Saccharomyces cerevisiae*	57.1 (8)	-	-	-
*Candida laurentii*	42.8 (6)	-	-	-
*Candida lusitaniae*	-	46.1 (6)	-	-
*Candida glabrata*	-	38.4 (5)	-	-
*Candida rugosa*	35.7 (5)	-	-	-
*Candida tropicalis*	-	23.1 (3)	-	-
*Candida pelliculosa*	-	-	15.3 (2)	40 (2)
*Candida parapsilosis*	14.3 (2)	-	-	20 (1)
*Candida sake*	-	-	-	60 (3)
*Rhodotorula glutinis*	21.4 (3)	-	-	20 (1)
*Rhodotorula mucilaginosa*	-	-	-	40 (2)
*Trichosporon asahii*	21.4 (3)	-	-	-
*Cryptococcus laurentii*	14.3 (2)	-	-	-

**Table 3:** Mold species isolated by cheese sauce, basil pesto, mushroom sauce and walnut sauce samples.

Isolated Molds	Samples (No.)			
	Cheese Sauce (14)	Basil Pesto (13)	Mushroom Sauce (13)	Walnut Sauce (5)
	% Contaminated Samples (No.)			
*Penicillium* spp	21.4 (3)	-	-	80 (4)
*Aspergillus* spp	1.2 (2)	76.9 (10)	61.5 (8)	60 (3)
*Aspergillus niger*	-	30.7 (5)	53.8 (7)	20 (1)
*Aspergillus flavus*	14.3 (2)	38.4 (4)	15.4 (2)	20 (1)
*Aspergillus ochraceus*	-	23.1 (3)	-	20 (1)
*Cladosporium herbarum*	7.1 (1)	61.5 (8)	-	60 (3)
*Mucorales*	7.1 (1)	38.5 (5)	-	40 (2)
*Alternaria alternata*	-	53.8 (7)	-	20 (1)
*Fusarium oxysporium*	-	30.77 (4)	-	20 (1)
*Paecilomyces* spp	-	-	-	20 (1)
*Scopulariopsis brevicaulis*	-	-	-	20 (1)
